# Anthropomorphic optical phantom of the neonatal thorax: a key tool for pulmonary studies in preterm infants

**DOI:** 10.1117/1.JBO.25.11.115001

**Published:** 2020-11-17

**Authors:** Andrea Pacheco, Haiyang Li, Monisha Chakravarty, Sanathana Konugolu Venkata Sekar, Stefan Andersson-Engels

**Affiliations:** a Tyndall National Institute, Biophotonics@Tyndall, Irish Photonic Integration Centre, Lee Maltings, Dyke Parade, Cork, Ireland; b University College Cork, Department of Physics, College Road, Cork, Ireland

**Keywords:** anthropomorphic optical phantom, gas spectroscopy, absorption, scattering, diffusive media

## Abstract

**Significance:** Gas in scattering media absorption spectroscopy (GASMAS) is a technique for gas sensing in cavities surrounded by scattering materials. GASMAS could be translated to the clinic to monitor lung function continuously and noninvasively in neonates. Accurate tissue phantoms are essential to assess the strengths and limitations of gas spectroscopy in gas-containing cavities in the human body.

**Aim:** The aim is to develop a detailed protocol to produce a long-lasting, multistructure tissue phantom of the thorax of a neonate. The phantom mimics the geometry and the optical properties of the main organs of the thorax and has an empty pulmonary cavity that facilitates GASMAS monitoring of gas content.

**Approach:** The anatomic geometry of heart, lungs, bones, muscle, fat, and skin was obtained from a neonatal computed tomography scan. Once segmented, organs were 3D printed and used to create negative rubber molds. The entire thorax was built in phantom material (silicone as matrix, black ink as absorber, and silica microspheres as scatters) by placing all phantom organs inside the muscle structure. Our phantom recipe was customized by mixing specific ratios of ink and spheres to match the optical properties of the different organs that were consider to be homogeneous.

**Results:** An anthropomorphic thorax phantom with the desired optical properties ($\mu_{a}$ and $\mu_{s}^{\prime }$) at 760 nm was built and used to obtain “transdermal” GASMAS measurements of oxygen content within the lung cavity.

**Conclusion:** A protocol to build a robust optical phantom of the thorax of a neonate was used to conduct benchtop studies. This recipe can be implemented to reproduce the geometry and optical properties of any human or animal tissue.

## 1 Introduction

Over the last decade, the feasibility of the clinical translation of gas in scattering media absorption spectroscopy (GASMAS) to measure the existence and concentration of gas in the human body has been pursued.^1^ There are various biomedical applications for this technique, such as detection of water vapor in necrotic femoral heads,^2^^,^^3^ molecular oxygen and water vapor within the maxillary and frontal sinuses,^4^ water vapor in the intestines of neonates, and air content in the lungs of infants using near-infrared (NIR) spectroscopy.^5^^–^^8^ The assessment of air content in the lungs of neonates has drawn attention in the NIR spectroscopy field due to the nature of lung tissue encompassing the air-filled alveoli.

Tissue-simulating phantoms are typically used for standardization, quality control, calibration, and validation of system performance.^9^ Studies of optical tissue phantoms that closely resemble a real clinical situation are an essential step in developing novel optical technologies,^10^^,^^11^ especially for vulnerable patients such as preterm neonates. The geometry of the phantom and materials that mimics the absorption and scattering properties of human tissue are chosen according to the intended use. To accurately characterize clinical devices in biophotonics, there is a need to develop anthropomorphic phantoms that mimic both optical properties and morphological features of human organs.^12^^–^^14^ 3D printing techniques have been used to replicate complex anatomic structures, such as human breast.^15^ However, reduced scattering and absorption properties of 3D-printed phantoms are, so far, restricted to the mixing compatibility of the printing material with scattering particles and ink. Most of the 3D printers just print one material at a time, making it impossible to obtain a phantom with diverse optical properties in a single cross section.

Molding tissue layers with accurate optical properties of interest in diffuse optics has been achieved.^16^ However, in cases such as GASMAS studies where the focus is on respiratory health care of neonates, the phantoms must mimic properties of the main thoracic organs and facilitate gas exchange inside the lungs. This issue was solved by Larsson et al.^17^ who built a 3D optical phantom of the thorax of a preterm infant. The model consists of void white nylon shells ($\mu_{a}=0.02\;{\mathrm{cm}}^{-1}$ and $\mu_{s}^{\prime }=46.5\;{\mathrm{cm}}^{-1}$) in the shape of skin, bone, and heart, which are filled with liquid optical phantom materials of the corresponding organs. Another void shell filled with gas has the geometry of the lungs. In this thoracic model, the high scattering of the nylon interphases biases the propagation of light along the different organs. Typically, Intralipid-based tissue phantoms are employed. These phantoms are stable as long as the Intralipid is diluted with either purified water, demineralized water, or distilled water.^18^ However, liquid phantom preparations remain homogeneous for a period of hours.^9^ To reuse the solutions over many days, the storage and mixing process must be consistent and carefully conducted to ensure repeatability and homogeneity in the phantom optical properties.

We present, to the best of our knowledge, the first solid long-lasting anthropomorphic thoracic phantom with specifically defined homogeneous optical properties for different tissue structures and a pulmonary cavity that can be filled with various gas compositions. This phantom is key in the validation studies to address the clinical translation of GASMAS technology. The 3-D printing and molding method presented here is, however, not limited to anthropomorphic phantoms for GASMAS measurements but is general and could be translated to mimic any part of the human body for diverse optical imaging and sensing techniques.

## 2 Methods

**Fig. 1:**
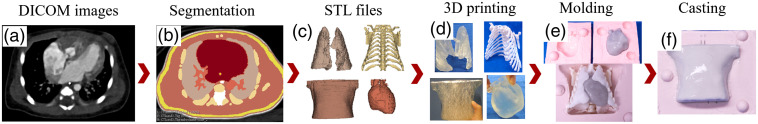
Workflow to build an anthropomorphic thorax phantom with optical properties of relevant organs and empty pulmonary cavity. (a) A stack of DICOM images from a CT scan of a neonate was used for (b) segmentations of all organ types. (c) Files for 3-D printing were created for each organ by segmenting the CT image stack. (d) These files were then 3-D printed to produce resin structures. (e) Siliglass mixed with silica microspheres and ink was molded to create a phantom with the specific homogenous optical properties of each organ. (f) Finally, organs were assembled to create the anthropomorphic optical phantom of the thorax of the neonate.

Figure 1 illustrates the workflow to construct the anthropomorphic optical phantom for the validation of the GASMAS technique, which can be used to assess the lung function in preterm neonates. This procedure is applicable to any tissue volume containing several structures with various optical properties by implementing the appropriate modifications. An optical phantom of a thorax of a 3.7-kg neonate was used as an example in this paper.

Briefly, anthropomorphic computer models of the relevant organs within the thorax were created after segmenting the organs in a computed tomography (CT) scan. All geometries were 3-D printed in resin. Afterward, rubber molds of the heart, lungs, and muscle were made.

The recipe for the silicone phantoms (heart, muscle, skin, and fat) was prepared assuming the optical properties of each organ to be homogeneous. Scatters and absorbers were uniformly mixed within the silicone matrix and the characteristic $\mu_{a}$ and $\mu_{s}^{\prime }$ were assigned based on the values available for each organ in literature. A solid silicone phantom with the optical properties of the heart was cast using the heart mold. This was cast along with the lungs and trachea models, which were made of coconut oil instead of silicone. The coconut oil has a liquid form when heated above 24°C; it was melted and poured in the molds. The molds containing the coconut oil were placed inside the refrigerator at 5°C for solidification. The solid structure with the pulmonary geometry enabled the correct placement of the innermost structure of the neonatal thorax (the heart), surrounded by the lungs connected to the main branches of the trachea. The parts were assembled according to the anatomy of the neonate by placing the resin bone structure around the lungs and incorporating the silicon phantom with the muscle optical properties to embed the above-mentioned organs.

Finally, two additional layers of the phantom, which match the optical properties of fat and skin, respectively, were added on top of the muscle phantom. The void pulmonary cavity required for the intended measurements was achieved by draining the coconut oil from the lungs and the main branches of the trachea. This empty chamber with the geometry of the trachea connected to the lungs can be filled with different gases for further GASMAS studies.

### 2.1 Segmentation: Acquisition of Organ Structure

**Fig. 2:**
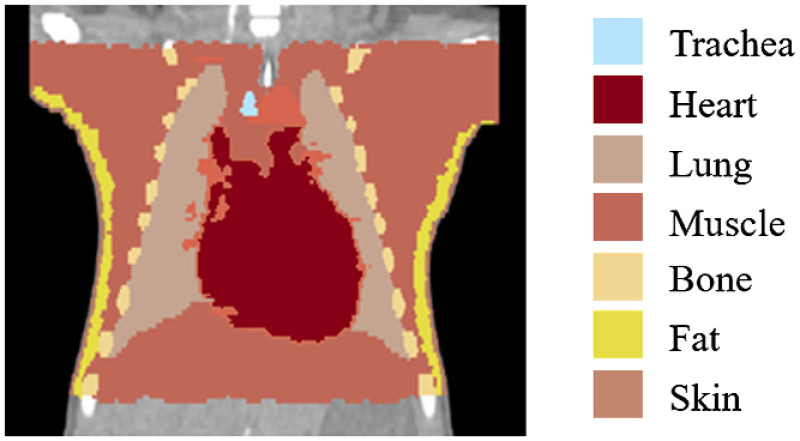
Segmentation of a DICOM image from a pseudoanonymized CT scan of a neonate, where the main organs (lungs, heart, muscle, bone, fat, and skin) have been identified and color coded.

The realistic geometry of the main organs in the thorax was achieved by segmenting Digital Imaging and Communications in Medicine (DICOM) images from a pseudoanonymized full-term female neonate (weight 3.7 kg). The segmentation was conducted by employing the NIRFAST software package, which enables the visualization of different organs recovered from overlaid 3-D standard medical images.^19^ The clinical indication for the CT scan was a heart dysfunction. There were 255 slices in the image for a body length of 22.3 cm. This means a z-resolution of 0.87 mm. The lateral resolution was similar. Motion artifacts were not observed in the scan, giving an accurate anatomical map of the body. The main thoracic organs (lung, trachea, muscle, bone, heart, fat, and skin) were shaded with different colors, slice by slice, until the whole geometry of each organ was color coded, as seen in Fig. 2. A computer 3-D stereo lithography (STL) file was created for each organ. The STL file for each organ was imported into Autodesk MESHMIXER software to enable smoothing. Since the files were created from a scan with coarse spatial resolution, the smoothing improved the file’s resemblance to real organs.^20^ The same software was used to increase the size of the trachea (to facilitate the removal of coconut oil content in the lung cavity) and to stitch the different segments of bone to print vertebrae, ribs, and shoulder blades as a singular structure.

### 2.2 3D Printing and Molding

**Fig. 3:**
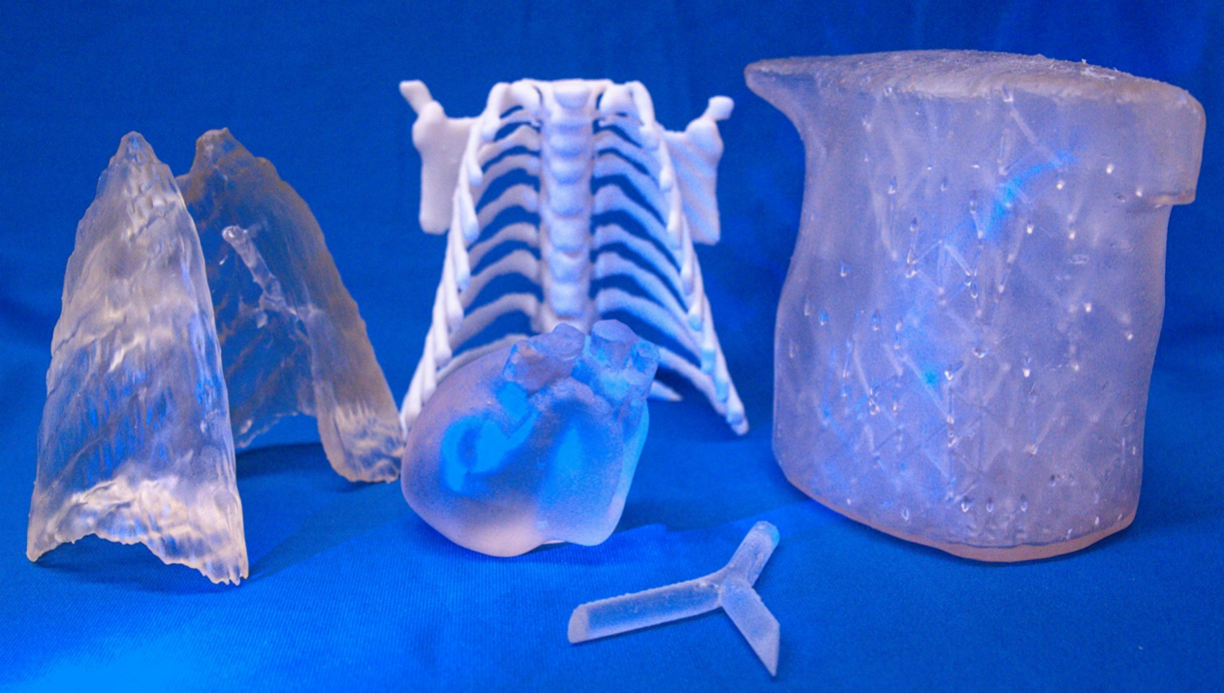
3D-printed resin structures of the organs in a neonate’s thorax to be used for molding, except for the bones (in white)- which were included in the phantom.

The smoothed STL files of the lungs, trachea, heart, muscle, and bone were 3D printed to scale with Form 2 (commercial 3D printer from Formlabs), as shown in Fig. 3. The printer operates with a violet light curing technique using a 405-nm laser to cure the resin layer by layer with an axial resolution of 25  μm. The biggest volume that can be printed with this printer model is $145\times 145\times 175\;{\mathrm{mm}}^{3}$. The dimensions of the thorax used in this study are below this limit, but for other applications, the build volume of the 3D printer can pose a constraint.

The bone structure was 3D printed in white resin from the company Formlabs. This material was characterized using a broadband time-resolved diffuse optical spectrometer, which was designed and validated to measure absorption and scattering spectra of highly diffusive media over 600 to 1350 nm.^21^ The measured absorption and reduced scattering coefficients of the resin were $\mu_{a}=0.02\;{\mathrm{cm}}^{-1}$ and $\mu_{s}^{\prime }=34.3\;{\mathrm{cm}}^{-1}$, respectively; these values differ from the bone optical properties published in literature $\mu_{a}=0.01\;{\mathrm{cm}}^{-1}$ and $\mu_{s}^{\prime }=9.3\;{\mathrm{cm}}^{-1}$.^22^ Despite these discrepancy, the bone structure was used as printed to facilitate the building of the complex thorax phantom, as it is used to keep in place the inner phantom organs.

The 3D-printed resin structures of lungs, trachea, heart, and muscle were used to create negative casting molds with Mold Max™ 30, a two-part silicone rubber compound manufactured by Smooth-On, Inc.

For the construction of the molds, square cardboard containers were constructed according to the dimensions of the lungs, trachea, muscle, and heart. The mold was made in two parts so that the cast could be removed later. For all the aforementioned resin organs, a 1-cm-thick support made of sponge was placed on the bottom of the respective box. The resin structures were daubed by a layer of Vaseline and placed on top of the sponge. The two compounds of the rubber (silicone and hardener in a 100:10 ratio) were mixed homogeneously and poured inside the containers to cover half of the volume of the organ structures. Spherical objects were also daubed with Vaseline and fixed on the surface of the liquid rubber to cast a lock system on the edge of the rubber mold, which guaranteed that the two parts of each mold will bind and remain fixed during casting.

The containers were placed in a vacuum degassing chamber for 15 min to eliminate any entrapped air in the pourable Mold Max™ rubber. The molds were then allowed to cure overnight (minimum 16 h) at room temperature. Afterward, the spherical objects were removed and a layer of Vaseline was spread over the cured rubber surfaces. A new mix of liquid rubber was poured on top until each of the resin structures was covered. Again, the rubber was allowed to cure overnight until the cardboard containers could be safely removed, and the two parts of each mold were pulled apart. The molding forms for casting were then completed.

To cast the different organ phantoms, a funnel was inserted through an x-shape incision made on the upper side of the molds. The preparation of the cast was done using a phantom recipe, which uses PlatSil SiliGlass as the matrix, silica microspheres as scatters, and ink as absorbers (Table 2). In the preparation of the recipe, cylindrical slabs of identical phantom material were produced to allow characterization of the optical properties through time-of-flight measurements.^23^ Table 1 provides the values of the aimed absorption and reduced scattering coefficients of each organ phantom for this study.

**Table 1:** The aimed optical properties taken from the literature at 760 nm for the different phantoms to be embodied in the thorax model.

Organ	$\mu_{a}$ (${\mathrm{cm}}^{-1}$)	$\mu_{s}^{\prime }$ (${\mathrm{cm}}^{-1}$)
Heart^22^	0.11	4.45
Muscle^22^^,^^24^	0.20	12.8
Fat^25^	0.07	13.2
Skin (dermis)^22^^,^^26^	0.03	24.8
Bone^22^	0.10	9.3

**Table 2:** Cast volumes and quantities of scatters and absorbers used in the preparation of each organ in the phantom thorax to match the optical properties of Table 1.

Organ	Volume (±0.05 ml)	Ink solution (±0.05 ml)	Silica microspheres (±0.05 g)
Heart	111.4	13.14	2.16
Muscle	608.6	43.89	30.22
Fat	53.5	5.49	5.27
Skin (dermis)	31.5	0.95	6.12
Lungs and trachea (cast with coconut oil)	195.7	—	—

Table 2 shows the volumes of the cast organs and the respective quantities of scatters and absorbers used for the preparation of each phantom recipe.

**Fig. 4:**
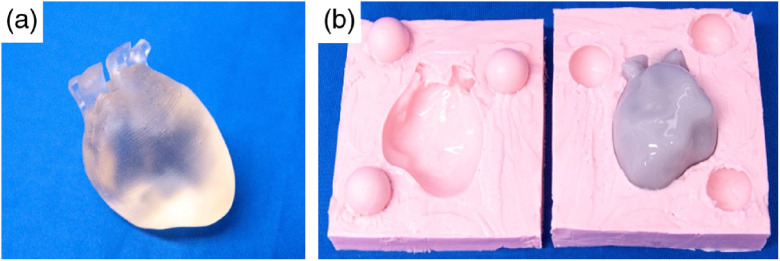
(a) 3D-printed resin heart structure. (b) Molded heart phantom with realistic optical properties defined in Table 1 (gray), sitting in the respective rubber negative cast (pink).

Figure 4 shows an example of the 3D-printed resin structure for the heart (a) followed by the respective negative cast (in pink) containing a molded silicone phantom (b). The lock system created with the three spherical inclusions is clearly seen.

### 2.3 Thorax Reconstruction and Void Pulmonary Cavity

**Fig. 5:**
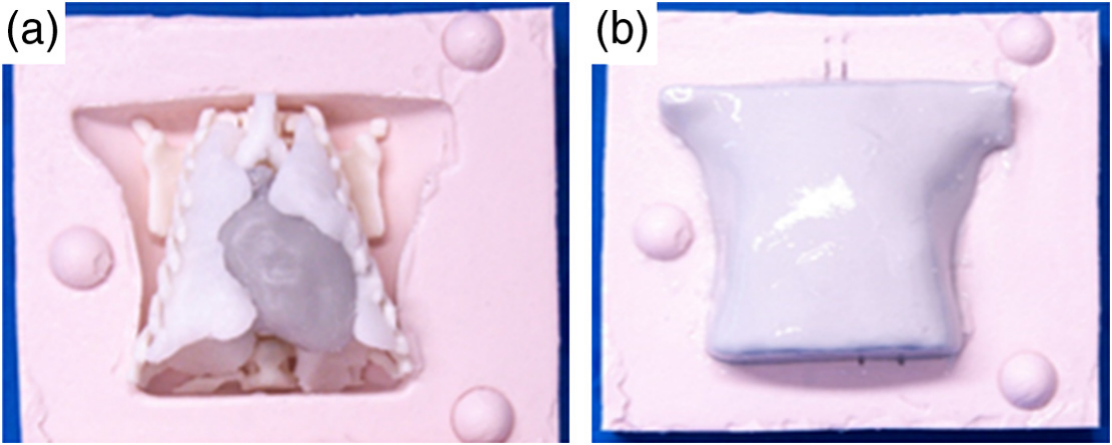
(a) Phantom bone, heart, lungs, and trachea placed in the lower side of the muscle mold before the mold was closed and filled with phantom material mimicking muscle tissue. (b) The same mold after curing the muscle phantom material,- where all tissues are assembled.

The complete thorax phantom was constructed by placing all the organs according to the structure of the thorax. The inner organs (heart and lung) were supported by the bone structure as can be seen in Fig. 5(a). After casting the silicone phantom with the muscle optical properties, all assembled organs fit together [Fig. 5(b)]. This is a great advantage because the path of the light inside the phantom would not be affected by the presence of boundary material among organs, solving one of the problems present in nylon phantoms.^17^ A phantom designed for GASMAS studies should ideally have no air pockets besides the air content cavity under study. In the case of the thoracic model, air should be present only in the pulmonary cavity. Dead space is avoided when manufacturing the phantom. Therefore, it is also a benefit for our application to ensure there are no gases outside the lung cavity within the phantom, to recreate the clinical scenario of gas presence only in the lungs of neonates.

To build the pulmonary cavity, the lungs and trachea were molded with coconut oil. After the silicone with muscle optical properties was cured, the phantom was heated above 24°C to melt the oil. The oil keeping the geometry of the trachea and lungs was poured out of a 5-mm hole pierced from the upper surface of the phantom reaching into the trachea, and the cavity was washed multiple times with acetone to remove any residual oil. This left inside the phantom an empty cavity with the correct pulmonary geometry form in which the gas composition can be measured using GASMAS technology. Two layers with fat and skin optical properties were poured over the cast muscle phantom to mimic the outer tissues of the thorax.

### 2.4 GASMAS Measurements for Phantom Validation

GASMAS is a nonimaging technique used to sense gas enclosed in a bulk scattering media. It exploits the differences in the features of absorption spectra between gases and condensed matter (liquid and solid) such as the width of the absorption bands, which are many orders of magnitude broader for condensed matter compared to gases, typically in the order of 10 nm for tissue versus 0.001 nm for gases. In a typical GASMAS measurement, the walls of the bulk material are illuminated and the light scatters into the gas cavity. Photons scattered back from the gas cavity are sensed with a photodetector placed over the bulk surface either in remittance or transmission geometry.^1^

The absorption signal from the gas is identified from the detected light intensity and the Beer–Lambert law is used to calculate the concentration of the gas. The Beer–Lambert law states that the intensity of the light (I), propagating through a gas with concentration (c) and along an optical path length (l), decays in an exponential way. The strength of the absorption also depends on the absorption cross section of the gas (ε), which is specific for each gas and the transition chosen for the absorption measurement.

$$I=I_{0}e^{-\varepsilon cl}.$$

In cases where the aim is to measure the gas concentration and the optical path length is unknown, a dual-laser source system is used to interrogate a reference gas (present in the same cavity) with known concentration. This can be done only if the wavelengths used to interrogate each of the gases are close enough to assume that the path lengths are the same.^27^

MicroLab Dual $O_{2}/H_{2}O$ is a GASMAS benchtop system produced by GASPOROX AB. This system has two diode lasers that sense oxygen (760 nm) and water vapor (820 nm). The wavelengths were chosen to be spectrally close so the absorption lines have a similar distribution of the light, and the absorption signal from water vapor can be used for normalization. Water vapor is used as a reference gas. Its concentration at a given temperature is calculated using the Arden Buck equation for a given temperature.^28^ Consequently, a value for the path length is obtained and used to estimate the oxygen concentration.

When measuring with the MicroLab Dual, both laser wavelengths are scanned across the absorption lines of oxygen (760 nm) and water vapor (820 nm) by alternating the respective currents. A light intensity dip is detected due to the absorption of the gas molecules present between the light source and the detector. The detection is improved using wave modulation spectroscopy (WMS), which generates as output the characteristic WMS amplitude signal in a GASMAS experimental set up.^5^

## 3 Results

We present an anthropomorphic phantom with the structure of the thorax of a neonate. The multiple organs were made of homogeneous phantom material, considering that each organ constitutes a single tissue type. In this case, we are not taking into account the presence of multiple structures in any human body organ, such as arteries, collagen, etc. The obtained optical properties of the different organs at 760 nm of the phantom built for this study are listed in Table 3. The measurements of the recipe were performed with three repetitions and the coefficient of variation (CV) was found to be less than 3%.^23^ The white resin used to 3D print the bone structure was 50 times less absorptive and 3 times more scattering than human bone. The rigid bone structure is needed to keep the phantom organs in place when curing the muscle, which simplifies the thorax building process and avoids the need for additional support structures.

**Table 3:** Optical properties of the organs at 760 nm embodied in the realistic thorax phantom. (The CV of these values is <3%.)

Organ	$\mu_{a}$ (${\mathrm{cm}}^{-1}$)	$\mu_{s}^{\prime }$ (${\mathrm{cm}}^{-1}$)
Heart	0.110±0.003	4.45±0.09
Muscle	0.200±0.005	12.8±0.3
Fat	0.070±0.002	13.2±0.3
Skin (dermis)	0.030±0.001	24.8±0.5
Bone (white resin)	0.020±0.001	34.3±1.4

The presence and concentration of molecular oxygen inside a reference box and the phantom was measured with MicroLab Dual $O_{2}/H_{2}O$.

**Fig. 6:**
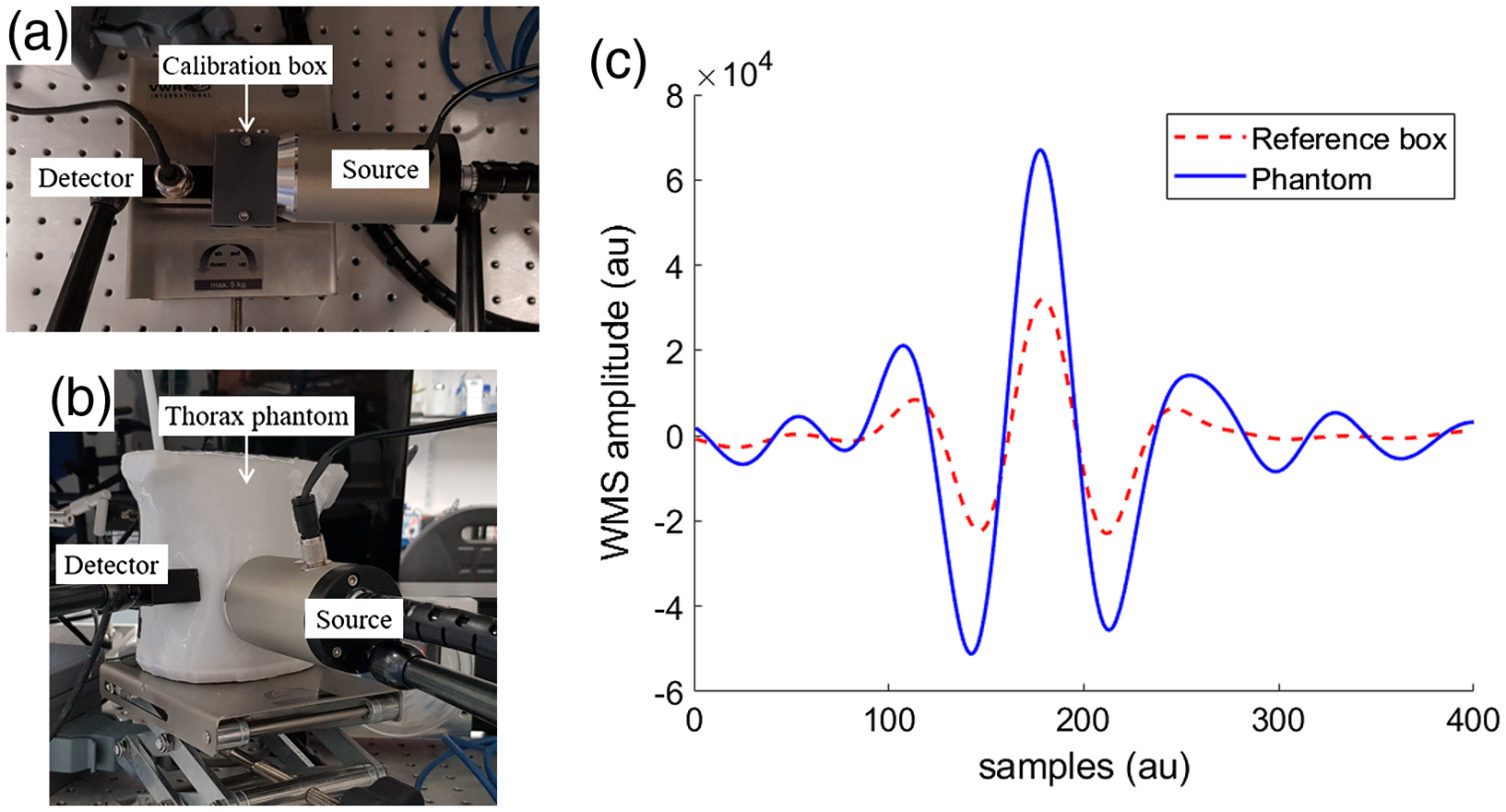
(a) MicroLab Dual $O_{2}/H_{2}O$ system with light source and detector placed on both sides of a reference box in transmission geometry. (b) MicroLab Dual $O_{2}/H_{2}O$ system with light source and detector placed over the thorax of the realistic phantom in remittance geometry used to sense the absorption signal of oxygen. (c) WMS amplitude signals produced by the absorption of molecular oxygen inside a reference box and the optical thorax phantom in transmittance and remittance geometry, respectively.

The dashed plot in Fig. 6(c) corresponds to the oxygen absorption imprint of the gas inside a 3-cm-width hollowed reference box in transmission geometry [Fig. 6(a)]. A signal with a similar characteristic absorption peak of oxygen was also obtained using the anthropomorphic phantom in remittance geometry [Fig. 6(b)]. The thicknesses of the thorax wall (between the skin layer and lung cavity) where the source and detector are placed are 6.4 and 19.3 mm, respectively. The plots of gas absorption correspond to the room oxygen present inside the box and phantom cavity. Transparent ultrasound gel was placed between the box and phantom surfaces and the probes (light source and detector) to ensure that the measured oxygen absorption signal corresponded to the gas inside the cavities.

A set of 10 measurements was taken with the reference box in transmission geometry (22.5°C) and with the phantom in remittance geometry (23.2°C), giving average oxygen concentrations of 19.2 % and 18.8%, with standard deviations of 0.01 and 0.03, respectively. Hence, we demonstrate the potential use of these realistic phantoms for quality control and validation of the GASMAS technique.

## 4 Discussion and Conclusion

A protocol to build an anthropomorphic phantom of the thorax of a neonate is presented. The phantom includes the geometrical structures of heart, muscle, fat, and skin together with the respective realistic homogeneous optical properties. If a different wavelength is chosen to perform future studies, a phantom recipe with different concentrations of scatters and absorbers can be prepared to cast another thorax phantom matching the optical properties of biological tissue at that wavelength. The geometry can be replicated reusing the organ molds from this study.

The multiple fine structures present in each organ, such as arteries and blood vessels, are not included. Each phantom organ is built with homogeneous optical properties.

The mismatch of optical properties of human bone and those of the bone structure of the phantom could be solved using a different 3D-printing material suitable for customized optical properties. One can also use a mold and cast the bone structure with the aimed optical properties with a material that is mechanically harder than SiliGlass, so the bone structure can still be used to keep the inner organs in place during the casting of the muscle tissue structure.

The experimental studies performed with this phantom resemble a simplified model of light propagation through the thorax of a neonate with gas absorption in the pulmonary cavity. The feasibility to conduct phantom studies with the MicroLab Dual $O_{2}/H_{2}O$ GASMAS benchtop system has been proven, raising the possibility to perform transdermal spectroscopy measurements of oxygen concentration inside the lung cavity of the phantom in a controlled environment. To the best of our knowledge, there is not an easy way to produce a phantom that accurately mimics simultaneously the absorption and scattering properties of human tissue at more than one wavelength. To perform further studies with the MicroLab Dual $O_{2}/H_{2}O$ GASMAS system, which operates with a dual source at 760 and 820 nm, we propose to build two geometrically identical phantoms, each with the optical properties matching the tissue at one of these wavelengths. Such studies constitute an important step in developing a lung function-monitoring technique for a vulnerable group of patients in the need for better tools in respiratory health care.

Further studies involving the injection of oxygen gas mixed with nitrogen and carbon dioxide in different concentrations inside the pulmonary cavity would be key to define the limitations of GASMAS technology in the development of a bedside clinical device for lung function assessment in neonates.

This recipe can be implemented to reproduce the 3D geometry and optical properties of any DICOM image file with multiple volumes from human or animal organ. As mentioned in Sec. 2.2, the only constraint is the maximum volume of the 3D printer, which is used to produce the solid physical models of the organs of interest. Even though PlatSil SiliGlass was chosen as the matrix for the phantom material in this study, other suitable phantom materials such as Sylgard might produce similar results as long as the pertinent changes are made with the curing requirements of the material.^29^

The present work opens a wide range of possibilities for complementary validation and standardization of diverse optical imaging systems and sensing techniques prior to prototyping of systems for clinical translation. Such phantoms may also be used to reduce some preclinical and clinical studies in the development of new photonics-based instruments or techniques.
